# A Narrative Review of Personal Protective Equipment Uses in Coronavirus Disease 2019 and Its Disposable Practices

**DOI:** 10.31662/jmaj.2020-0120

**Published:** 2021-04-02

**Authors:** Anil Giri, Binaya Sapkota, Ranish Shrestha, Asmita Priyadarshini Khatiwada, Rajib Tiwari, Manisha Aryal, Menaka Timilsina, Biplove Bhujel, Mohan Adhikari, Ranjit Sah, Divya Bhandari, Akihiko Ozaki, Cecilia Acuti Martellucci, Yasuhiro Kotera, Sayed Hamid Mousavi, Sunil Shrestha

**Affiliations:** 1 Pharmaceutical Sciences Program, School of Health and Allied Sciences, Faculty of Health Sciences, Pokhara University, Kaski, Nepal; 2 Department of Pharmaceutical Sciences, Nobel College, Affiliated to Pokhara University, Kathmandu, Nepal; 3 Infection Control Unit, Nepal Cancer Hospital and Research Center, Lalitpur, Nepal; 4 Department of Pharmaceutical and Health Service Research, Nepal Health Research and Innovation Foundation, Lalitpur, Nepal; 5 Department of Microbiology, Tribhuvan University Teaching Hospital, Institute of Medicine, Kathmandu, Nepal; 6 Medical Governance Research Institute, Tokyo, Japan; 7 Department of Breast Surgery, Jyoban Hospital of Tokiwa Foundation, Fukushima, Japan; 8 Department of Medical Sciences, University of Ferrara, Ferrara, Italy; 9 Human Sciences Research Centre, University of Derby, Derby, UK; 10 Medical Research Center, Kateb University, Kabul, Afghanistan

**Keywords:** coronavirus disease 2019 (COVID-19), healthcare waste (HCW) management, personal protective equipment (PPE), severe acute respiratory syndrome coronavirus 2 (SARS-CoV-2)

## Abstract

Since severe acute respiratory syndrome coronavirus 2 (SARS-CoV-2), responsible for causing coronavirus disease 2019 (COVID-19), is transmitted through close contact and droplets, people, especially those at risk of infection, must follow preventive measures in the community and healthcare settings. Healthcare personnel (HCP) must appropriately select and use personal protective equipment (PPE) with sensible donning and doffing and disposal practices. A narrative review of the existing literature was conducted, in which articles from Scopus, PubMed, Google Scholar, ScienceDirect, and Web of Science were collected. The primary findings of the retained articles were reviewed according to official recommendations on PPE use. The World Health Organization (WHO), US Centers for Disease Control and Prevention (CDC), and European Center for Disease Control and Prevention (ECDC) recommend standard precautions for contact transmission, respiratory transmission, and droplet precautions among HCPs caring for patients with COVID-19. Indeed, healthcare workers working in high-risk areas, as well as the public, when social distancing cannot be assured, must wear PPE such as face mask and protective eyewear to prevent the transmission of SARS-CoV-2 infection. Due to the increased use of PPE, the potentially infectious waste stream has been rapidly increasing, requiring safe and adequate solid waste management. The proper use of PPE and management of waste generated from COVID-19 care centers can reduce the risk of COVID-19 infection. These measures should be implemented to counter the rapid spread and any long-term impacts of the current pandemic.

## Introduction

The first human case of coronavirus disease 2019 (COVID-19) caused by a novel coronavirus (severe acute respiratory syndrome coronavirus 2 (SARS-CoV-2) was reported in Wuhan, China, in early December 2019. Coronaviruses have been responsible for several outbreaks, including the severe acute respiratory syndrome (SARS) pandemic of 2002-2003 and the Middle East respiratory syndrome outbreak of 2012, which originated in Saudi Arabia ^(1)^. A total of 101,406,059 COVID-19 cases and 2,191,898 deaths (as of January 30, 2021) around the world is reported by the World Health Organization (WHO) ^(2)^. While 81% of patients with COVID-19 have no symptoms or mild pneumonia, severe presentations, such as acute respiratory distress syndrome, occurs in 14% of patients ^(3), (4)^. Further, only 5% of patients present with respiratory failure, septic shock, and/or multiple-organ failure ^(4)^.

The human-to-human transmission of SARS-CoV-2 primarily occurs through direct, indirect, or close contact with infected people via respiratory secretions and saliva, or through their respiratory droplets, expelled when an infected person coughs, sneezes, talks, or sings ^(5), (6)^. Airborne transmission can also occur, although less frequently ^(7)^. As COVID-19 is primarily transmitted through close contact and droplets, those coming in contact with the infected or those at risk of infection must follow preventive measures in community and healthcare settings. Effective preventive measures in the community include maintaining hand and respiratory hygiene; avoiding touching one’s eyes, nose, and mouth; wearing a face mask; and maintaining at least two-meter distance from others, especially those who exhibit symptoms. In addition, healthcare workers must appropriately use personal protective equipment (PPE) (selecting the right PPE with proper donning and doffing and disposal of the used ones) when coming in contact with suspected or confirmed cases of COVID-19 ^(8)^. Therefore, this study aims to discuss the use of PPE and its disposal practices during the COVID-19 pandemic.

## Method

The present review focuses on the use of PPE as recommended by guidelines and its disposal practice after contact with suspected or active COVID-19 case. The databases of Scopus, PubMed, Google Scholar, ScienceDirect, and Web of Science were searched using the following search keywords: “COVID-19” OR “Personnel Protective Equipment” OR “PPE” OR “Disposal Practice” OR “PPE Disposal Practice” OR “PPE Guidelines” OR “Hospital waste.” The PPE use guidelines published by the WHO, US Centers for Disease Control and Prevention (CDC), and European Center for Disease Control and Prevention (ECDC) were included in this review. The PPE use guidelines of organizations beside those mentioned above were excluded. Articles containing original data on PPE use practices on community, hospital, and COVID-19 care center and articles on healthcare waste (HCW) disposal and management practices on community and hospital setting in context of COVID-19 were included in the review. Furthermore, PPE use and disposal of general practices were excluded. Articles published in the English language were included. The articles were selected based on relevance to COVID-19 and PPE use and disposal practice. Articles published from December 1, 2019, to October 5, 2020, were included for analysis purpose.

## Results

**Figure 1.**
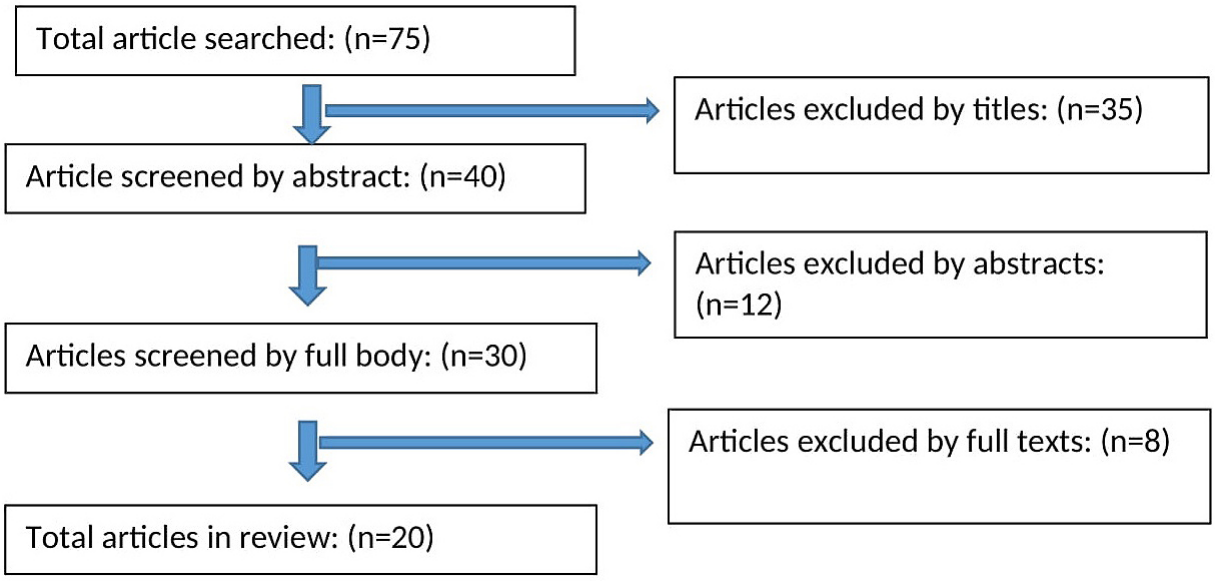
Selection of articles for review.

Table 1 shows comparison of PPE guideline recommendations by the WHO, CDC, and ECDC. Precautions against COVID-19 recommended by the WHO, CDC, and ECDC were summarized. A total of 75 articles were initially identified and 20 full texts were screened to verify inclusion and exclusion criteria, and 5 articles were retained for final review (Figure 1).

**Table 1.** Comparison of Personal Protective Equipment (PPE) Guideline Recommendations by the World Health Organization (WHO), Centers for Disease Control and Prevention (CDC), and European Center for Disease Control and Prevention (ECDC).

Setting/time issued	WHO (March 2020)	CDC (May 2020)	ECDC (March 2020)
Contact and droplet precautions	Medical mask	N95 or higher-level respirator	FFP2 or FFP3 respirator
	Eye protection (goggles) or facial protection (face shield)	Eye protection (e.g., goggles, face shield)	Eye protection
	Clean, nonsterile, long-sleeved gown, gloves, and boots	Gloves	Gloves
		Isolation gown	Long-sleeved, water-resistant gown
Airborne precautions for aerosol-generating procedures	NIOSH-certified N95		
	EU standard FFP2 /FFP3 or equivalent		
	Eye protection (i.e., goggles or a face shield)	N95 or higher-level respirator	FFP3 respirator
	Clean, nonsterile, long-sleeved gown	Eye protection	Eye protection	
		Gloves	Gown
	Waterproof apron (if gowns are not fluid-resistant)	Gloves	Gloves
Collecting specimens (aerosol-generating procedures not included)	Medical mask	N95 respirator (or higher-level respirator) or face-mask (if a respirator is not available)	Surgical mask (if available, FFP2 respirator)
	Eye protection	Eye protection	Eye protection
	Gown	Gown	Gown
	Gloves	Gloves	Gloves
			Drive-through or outdoor facilities
			Surgical mask (in addition to gloves, goggles, and gown)
Inpatient care	Medical mask	N95 respirator (or higher-level respirator) or face-mask (if a respirator is not available)	FFP2 respirator
	Eye protection	Eye protection	Goggles or face shield
	Gloves	Gown	Gown or apron
	Gown	Gloves	Gloves

## Current Recommendations for PPE Use During the COVID-19 Pandemic

The PPE use practices and outcomes are listed in Table 2. The WHO recommends implementing droplet and contact precautions during nonaerosol-generating procedures of patients with COVID-19. Medical masks (surgical masks) and eye protection are recommended during direct care of patients to prevent droplet transmission. For contact precaution, long-sleeved, water-resistant gowns and gloves are recommended. Respirators such as N95 or filtering facepiece (FFP3) (standard or equivalent), gowns (aprons should be used if gowns are not fluid-resistant), gloves, eye protection, and aprons are recommended during aerosol-generating procedures on patients with COVID-19 (such as intubation, cardiopulmonary resuscitation, nebulization, and chest physiotherapy) ^(8)^. The CDC and ECDC recommend standard precautions for contact transmission, respiratory transmission, and droplet precautions among healthcare workers who look after patients with COVID-19 ^(9), (10)^. The WHO, CDC, and ECDC recommend rational use of PPE for healthcare personnel (HCP) when taking care of suspected patients with SARS-CoV-2 ^(8), (9), (10)^.

**Table 2.** Personal Protective Equipment (PPE) Use Practices and Outcomes.

Authors	Country	Findings/outcomes and comments
Kumar J ^(19)^	Pakistan	Health workers had a positive attitude but had moderate-to-poor level of knowledge and practice regarding face masks. Health workers and general public awareness campaigns regarding the proper use of face mask using all social media available resources would help this pandemic.
Lyu W ^(20)^	USA	In the USA, states mandating face masks in public produced a more significant decline in daily COVID-19 growth rates than states that did not issue such mandates.
Liu M ^(21)^	China	Frontline healthcare workers, provided with appropriate PPE for delivering health care to patients with COVID-19 (including aerosol-generating procedures) were found negative for SARS-CoV-2 nucleic acids and specific IgM and IgG antibodies.
Renaud PP ^(22)^	Canada	The knowledge and practice regarding PPE use among healthcare workers were found to be inadequate and improper. Only 50% identified the correct donning and 35% correct doffing. The majority (70%) identified the need to perform hand hygiene before removing face mask and/or eye protection.
Ong JJY ^(23)^	Singapore	The frequency of PPE use for healthcare workers increased during the COVID-19 emergency. The mostly used PPEs were N95 masks for respiratory system protection and goggles for eye protection. The majority of healthcare workers (81%) experienced PPE-associated headaches. The onset of headache was less than 60 min after donning the PPE and resolved within 30 min of PPE doffing.

Additionally, all staff within the hospital must adopt hand hygiene, environmental disinfection, and proper waste management. Workers who are not involved in any COVID-19-relevant activity need not use PPE but should strictly follow standard precautions, such as maintaining a minimum distance of 2 meters between individuals, among others ^(8), (9), (10)^.

In addition to the provision of PPE, one must also concentrate on their proper use, especially appropriate donning and doffing procedures to minimize biosafety breaches and potential exposure to HCP ^(11)^.

## Literature Findings

### HCW Management during the COVID-19 Pandemic

The overall waste generated was reduced due to lockdown implemented in several nations, but the HCW generated increased due to single-use PPE. Among the total waste, PPE, gloves, tissue papers, face masks, and boots accounted for 10.8%, 1.7%, 10.0%, 1.6%, and 12.2%, respectively ^(12)^. Traditionally, PPE was predominantly used in the hospital environment, but because of the COVID-19 pandemic, PPE is widely used in community and household settings as well. Owing to the increased use of PPE, the potentially infectious waste in domestic solid waste streams has risen, urgently necessitating safe and adequate management of solid waste ^(13)^.

The WHO (July 29, 2020) published a guideline on water, sanitation, hygiene, and waste management for SARS-CoV-2, which included measures to be taken while managing HCW. HCWs generated from COVID-19 care centers are not different from other HCWs, and no additional waste management recommendation is needed ^(12)^. Nevertheless, the provision of safe water, sanitation, waste management, and hygiene conditions is required for infection prevention from HCWs. Potential infectious wastes, such as sharps, bandages, and pathological waste, must be safely collected and segregated, treated on-site, and then disposed of. Reusable PPE, such as utility gloves, heavy-duty gloves, and reusable plastic aprons, are cleaned with soap and water and then decontaminated with 0.5% sodium hypochlorite solution each time they are used, whereas single-use gloves and gowns must be discarded as infectious waste after each use ^(12)^.

In the developing country of Nepal, the Ministry of Health and Population published a guideline for HCW management in 2014 and COVID-related waste management in 2020. This guideline for disinfection delineates that reusable PPE must be washed with soap/detergent then autoclaved or soaked in 0.5% sodium hypochlorite solution for at least 30 minutes and finally washed with at least 10 times of the volume of clean water and dried before reuse. The guideline states that nonreusable PPE must be autoclaved or soaked in 0.5% sodium hypochlorite solution for 30 minutes and disposed of in a municipal landfill or burial pit ^(14)^.

During the COVID-19 outbreak in China, the disposal practice of HCPs has changed from the conventional technique to alternative techniques, such as from decentralization to centralization, from irregular to regular management, and from mostly incineration to non-incineration disposal technologies, such as steam (autoclave), dry heat, microwave sterilization, or chemical disinfection ^(15)^.

Disposable medical masks can be reused (during a shortage of masks) following disinfection with hydrogen peroxide vapor, ultraviolet or gamma irradiation, and ethylene oxide treatment ^(16)^. In many developing countries, specific techniques for the HCW management are absent. Thus, such waste is often disposed of alongside municipal solid wastes in the open or poorly managed landfills. Improper management of PPE while treating patients with COVID-19, such as using inadequate PPE or reusing them, has been shown to pose a significant risk for transmission ^(16), (17)^.

There are also instances of discarding single-use PPE, such as face masks and gloves, haphazardly in parking lots at the grocery stores, streets, and gardens. Such careless practice could increase the risk of transmission through waste materials and harm the environment since most of the PPE is made from nondegradable materials ^(18)^.

## Conclusion

COVID-19 is a pandemic that has taken lives of more than 2 million people all over the world as of January 2021. HCPs, especially those working in high-risk areas, and those looking after patients with COVID-19 must use all required PPE, and the public, when physical distancing is impossible, must wear PPE, such as face masks, to prevent possible transmission of SARS-CoV-2 infection. Taken together with the recommendations from the literature, it is concluded that the proper use of PPE and proper management of HCWs generated from COVID-19 care centers can reduce the risk of COVID-19 spread.

## Article Information

### Conflicts of Interest

Akihiko Ozaki received personal fees from MNES Inc., outside the submitted work.

### Author Contributions

All authors made substantial contributions to the conception and design and acquisition and interpretation of data, took part in drafting the article and revising it critically for important intellectual content, agreed to submit to the current journal, gave final approval of the version to be published, and agree to be accountable for all aspects of the work.

### Approval by Institutional Review Board (IRB)

Ethical issues (including plagiarism, informed consent, misconduct, data fabrication and/or falsification, double publication and/or submission, and redundancy) have been completely observed by the authors.
